# Elevated Allele Frequency of a Common Germline *LAG3* Variant Associated with Anemia, Thrombocytopenia and Peripheral Blast Percentage in Acute Myeloid Leukemia

**DOI:** 10.3390/cancers18101671

**Published:** 2026-05-21

**Authors:** Katja Seipel, Inna Shaforostova, Elisa Tarozzi, Marie-Noelle Kronig, Ulrike Bacher, Thomas Pabst

**Affiliations:** 1 Department of Medical Oncology, Inselspital, University Hospital Bern, University of Bern, 3010 Bern, Switzerland; innaivanovna.shaforostova@insel.ch (I.S.); elisa.tarrozzi@students.unibe.ch (E.T.); marie-noelle.kronig@insel.ch (M.-N.K.); 2 Department for Biomedical Research, University of Bern, 3008 Bern, Switzerland; 3 Department of Hematology, University Hospital Bern, 3010 Bern, Switzerland; veraulrike.bacher@insel.ch

**Keywords:** lymphocyte activation gene 3 (LAG3), cytotoxic T-lymphocyte associated protein 4 (CTLA4), single-nucleotide polymorphism (SNP), minor allele frequency (MAF), acute myeloid leukemia (AML), genetic risk

## Abstract

Background: Lymphocyte-activation gene 3 (*LAG3*) and cytotoxic T-lymphocyte-associated protein 4 (CTLA4) are immune checkpoint receptors and inhibitory regulators of T-cells. Methods: Here, we analyzed the prevalence and potential impact of the *LAG3* gene variant rs870849 and the *CTLA4* gene variant rs231775 in AML patients eligible for autologous stem cell transplantation. Results: While *CTLA4* rs231775 was prevalent at reduced minor allele frequencies (MAF 0.33), *LAG3* rs870849 was prevalent at elevated minor allele frequencies (MAF 0.58) in AML patients, compared to the allele frequencies in the European population (MAF 0.37 and MAF 0.39). The gene risk analysis indicated a dose-dependent risk of AML disease associated with *LAG3* rs870849, but no risk associated with *CTLA4* rs231775. Baseline blood count profiles differed across *LAG3* genotypes, suggesting a link between *LAG3* rs870849 and disease-associated levels of anemia, thrombocytopenia and peripheral blast percentage. Conslusions: The germline *LAG3* variant rs870849 may be associated with AML disease risk and specific hematological disease features.

## Simple Summary

Germline variants of immune-regulatory genes may affect immune checkpoint functions, disease susceptibility, disease features and treatment response in hematologic malignancies. This is the first report on the prevalence and impact of germline *LAG3* and *CTLA4* variants in AML. The *LAG3* gene variant rs870849 was prevalent at high allele frequencies, the *CTLA4* gene variant rs231775 at low allele frequencies in AML patients eligible for autologous stem cell transplantation. Genetic risk was associated with *LAG3* rs870849, but not with *CTLA4* rs231775. Baseline clinical characteristics of AML patients varied according to *LAG3* genotype indicating an association of *LAG3* rs870849 with erythrocyte, leukocyte and platelet imbalance in AML disease.

## 1. Introduction

There is accumulating evidence that the development of cytotoxic T cell dysfunction, especially exhaustion and senescence, contribute to disease progression in AML and other hematological malignancies [1,2,3]. Within the bone marrow microenvironment, antitumor immunity can be impaired through effector T-cell exhaustion and expansion of regulatory T cells [4]. Several immune checkpoint molecules—such as programmed death-1 (PD-1), cytotoxic T-lymphocyte-associated protein 4 (CTLA-4), and lymphocyte activation gene 3 (LAG3, CD223)—play pivotal roles in regulating these immune interactions [5]. Among these, LAG3 has emerged as a critical regulator of T-cell exhaustion and a promising target for next-generation tumor immunotherapies [5,6].

The *LAG3* gene, located on chromosome 12p13.31, encodes a type I transmembrane protein expressed on activated CD4+ and CD8+ T cells, natural killer cells, and dendritic cells [6]. LAG3 interacts with major histocompatibility complex class II (MHC-II) receptors and fibrinogen-like protein 1 (FGL1), delivering inhibitory signals that suppress T-cell proliferation and cytokine production [7]. Blocking LAG3 can restore T-cell cytotoxic activity and enhance anti-tumor responses, particularly when combined with PD-1 inhibition [3].

The functions of immune checkpoints such as CTLA4 and LAG3 can vary based on genetic variants in the respective genes. Single-nucleotide polymorphisms (SNPs) in the *LAG3* gene can influence transcription, splicing, and receptor structure, thereby altering immune tolerance thresholds [8]. For example, the common missense variant rs870849 (C > T; I455T) and the intron variant rs2365095 (A > G) have been associated with immune dysregulation and susceptibility to various diseases [9,10]. Polymorphisms in immune-regulatory genes such as *CD19*, *CTLA4* and *LAG3* have been reported to affect immune checkpoint functionality and influence treatment outcomes in hematologic malignancies [11,12,13]. Specifically, donor genotypes for *LAG3* variant rs870849 genotypes have been linked to adverse outcomes in allogeneic stem-cell transplantation, while other polymorphisms in *LAG3* have been associated with altered immune recovery in bone marrow failure syndromes [8,10].

Recent evidence suggests that LAG3 operates alongside other inhibitory receptors–PD-1, TIM-3, and TIGIT to establish co-inhibitory networks that tightly regulate immune activation and maintain tolerance [14]. In this context, germline variations in *LAG3* and *CTLA4* may serve as genetic predispositions for inadequate immune surveillance and tumor persistence. Furthermore, the expanding clinical use of combined PD-1/LAG3 blockade highlights the translational significance of targeting these pathways [15]. Improved understanding of *CTLA4* and *LAG3* polymorphisms could shed light on the pathogenesis of acute myeloid leukemia (AML) and support the development of tailored immunotherapeutic approaches.

This study focused on evaluating the risk associated with the common *CTLA4* SNP rs231775 and the *LAG3* SNP rs870849 in contributing to AML development, the potential impact on disease features, and the potential impact on clinical outcomes. By genotyping well-defined patient cohorts and analyzing the correlation of these genetic variants with clinical parameters, this research aims to clarify the role of *CTLA4* and *LAG3* genetic variability in AML susceptibility and treatment outcomes. Identifying risk-associated variants in these genes may enhance understanding of disease mechanisms and support the development of personalized immunotherapy strategies for AML patients.

## 2. Materials and Methods

### 2.1. Gene Sequence and Gene Risk Analysis

Genomic DNA was extracted from mononuclear cells (PBMCs) isolated from the peripheral blood of AML patients in complete remission after induction therapy. The *CTLA4* and *LAG3* genes were PCR-amplified and analyzed by Sanger sequencing as described [11,12]. Results of sanger sequencing were unambiguous for homozygosity of an snp (allele dosage equaling signal strength 2:0 or 0:2) and heterozygosity (allele dosage equaling signal strength 1:1). Gene characteristics were analyzed on the UCSC genome browser (https://genome.ucsc.edu/, accessed on 10 April 2026) [16]. Gene risk was analyzed using the gene association calculator (https://sites.google.com/view/generiskcalc/home/gene-association-calculator, accessed on 10 April 2026) [17]. The genealogical variant age was analyzed with GEVA (https://human.genome.dating/, accessed on 10 April 2026) [18].

### 2.2. Clinical Data

We carried out a retrospective study at Inselspital, University Hospital Bern, Switzer-land, focusing on a cohort of 140 patients diagnosed with acute myeloid leukemia (AML) between 2006 and 2023. The classification of AML at diagnosis was based on the French–American–British (FAB) system [19]. Patients were categorized into prognostic groups according to molecular genetics and karyotyping of their AML cells, following the guidelines from the European Leukemia Network (ELN), as outlined in ELN2017 and ELN22 [20,21]. Each patient underwent induction therapy, followed by high-dose chemotherapy (HDCT) and autologous stem cell transplantation (ASCT) in their first remission. This study adhered to the principles of the Declaration of Helsinki and received approval from the Ethics Commission of the Canton of Bern (decision number 2025-00853, date of approval 24 April 2025). Informed consent was obtained from all participants. Clinical data were analyzed as previously detailed [22].

## 3. Results

### 3.1. Characteristics of the CTLA4 rs231775 and LAG3 rs870849 Variants

rs231775 is a common *CTLA4* germline variants with a minor allele frequency (MAF) of 0.37 (ALFA) in the European population, indicating a 40% prevalence of AA homozygotes, 46% AG heterozygotes and 14% GG homozygotes. The encoded CTLA4 proteins are characterized by an amino acid variation at position 17, alanine or threonine, located in the leader peptide, associated with altered protein expression, processing and function. According to the UCSC genome database [16] CTLA4 T17 is the ancestral hominoid *CTLA4* variant, found in human, bonobo, chimp and gorilla, with an evolutionary age of 10 million years, while CTLA4 A17 is a human-specific *CTLA4* variant.

rs870849 is a common *LAG3* germline variant with MAF 0.43 (ALFA) in the European population indicating a 32% prevalence of CC homozygotes, 49% CT heterozygotes and 19% TT homozygotes. The two encoded LAG3 proteins are characterized by an amino acid variation at position 445, threonine or isoleucine, located in the transmembrane domain of the receptor, associated with altered protein expression, processing and function. According to the UCSC genome database [16] LAG3 I455 is the ancestral mammalian *LAG3* variant, found in human, mouse, dog, and elephant, with an evolutionary age of 100 million years, while LAG3 T455 is a hominoid *LAG3* variant, found in human, bonobo, chimp, and gorilla, with an evolutionary age of 10 million years.

### 3.2. Elevated Prevalence of LAG3 rs870849 in AML Patients

The sequences of exon one of the *CTLA4* gene and exon seven of the *LAG3* gene were analyzed in peripheral blood cells from 140 AML patients who achieved their first complete remission after induction therapy. Among these, 62 patients (44%) were found to have two major alleles encoding CTLA4 T17 (T17hom), while 64 patients (46%) carried one allele rs231775 (T17Ahet), and 14 patients (10%) carried two alleles rs231775 (A17hom). In the case of the *LAG3* gene, 28 patients (20%) possessed two major alleles encoding LAG3 I455 (I455hom), 63 patients (45%) had one allele rs870849 (I455Thet), and 49 patients (35%) carried two alleles rs870849 (T455hom).

**Figure 1:**
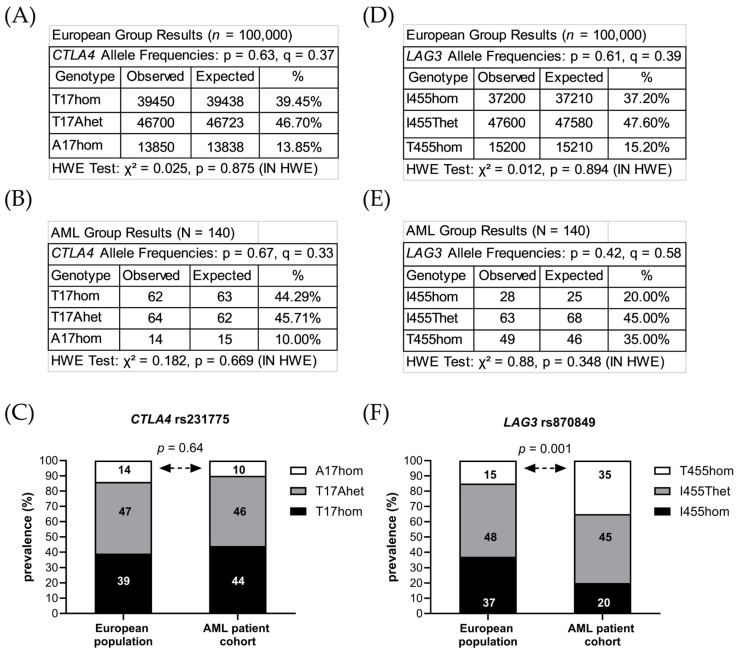
Prevalence of *CTLA4* rs231775 and *LAG3* rs870849 in the local AML cohort and the broader European population. Allele frequencies of *CTLA4* rs231775 in the European population (**A**) and within the local AML cohort (**B**). A comparative prevalence assessment of *CTLA4* rs231775 between the local AML cohort and the European population (**C**). Allele frequencies of *LAG3* rs870849 in the European population (**D**) and the local AML cohort (**E**). A comparative prevalence analysis of *LAG3* rs870849 in the local AML cohort versus the European population (**F**). Hardy–Weinberg Equilibrium (HWE) is referenced for genetic balance, with “p” denoting the major allele and “q” representing the minor allele.

Allele frequencies were calculated in the AML cohort and compared to the allele frequencies observed in the European population with rs231775 and rs870849 allele frequencies of 0.36 and 0.39, respectively (ALFA sample size > 500,000, https://www.ncbi.nlm.nih.gov/snp/, access date 7 May 2026). In the European population the minor allele frequencies of rs231775 and rs870849 averaged 0.37 and 0.39, respectively (Figure 1A,D). In the AML cohort, the minor allele frequencies of rs231775 and rs870849 averaged 0.33 and 0.58 (Figure 1B,E). The observed rs231775 frequencies (MAF 0.33) in the AML cohort were lower than the European control group (MAF 0.37) (Figure 1C). In contrast, the rs870849 allele frequencies were significantly higher in the AML cohort (MAF 0.58) compared to the European control group (MAF 0.39) (Figure 1F). The key inferences from a genetic association study may be compromised if Hardy–Weinberg equilibrium (HWE) is violated [23]. With no deviations from HWE in the analyzed dataset, the *LAG3* and *CTLA4* allele frequencies calculated for the AML groups were validated.

### 3.3. Genetic Risk Associated with LAG3 rs870849

**Figure 2:**
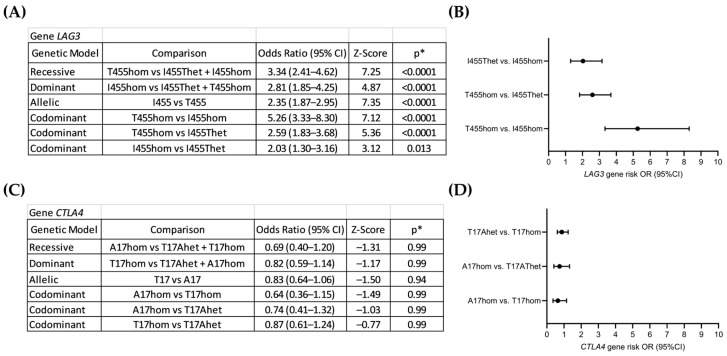
Genetic risk associated with *LAG3* rs870849 and *CTLA4* rs231775 in local AML cohort. Gene risk analysis of *LAG3* rs870849 (**A**,**B**) and *CTLA4* rs231775 (**C**,**D**). *LAG3* gene risk analysis in different genetic models (**A**). Forest plot of gene risk associated with *LAG3* rs870849 in the codominant model (B). *CTLA4* gene risk analysis in different genetic models (**C**). Forest plot of gene risk associated with *CTLA4* rs231775 in the codominant model (**D**). OR: odds ratio; CI: confidence interval. * *p* values with Bonferroni correction for multiple testing (α = 0.05/7 = 0.00714).

Genetic risk associated with the *LAG3* rs870849 and *CTLA4* rs231775 variants was analyzed using the Hardy–Weinberg equilibrium calculator in different genetic models including allelic, dominant, codominant and recessive [24]. *LAG3* rs870849 was significantly associated with risk for AML, in all genetic models, at odds ratios (OR) between 2 and 5.2 (Figure 2A,B). The gene risk associated with *LAG3* 870849 was dose-dependent with OR 5.2 in the codominant model at dosage 2 vs. 0, OR 2.6 at dosage 1 vs. 0, and OR 2 at dosage 2 vs. 1. In contrast, gene risk associated with *CTLA4* rs231775, in all genetic models, indicated inverse OR values between 0.64 and 0.87; however, these results were without significance (Figure 2C,D).

### 3.4. Clinical Characteristics According to LAG3 rs870849

**Figure 3:**
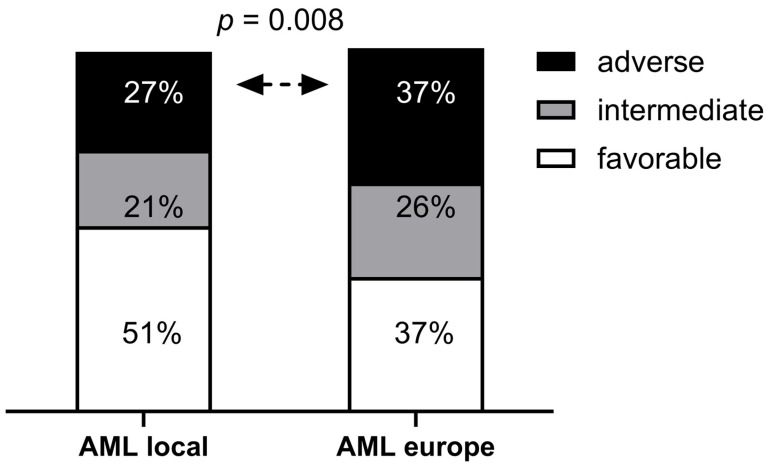
Selection bias in the studied AML cohort. Differences in the ELN distribution in the local AML cohort compared to an unbiased European AML cohort. The local AML cohort was selected for treatment setting with consolidation therapy in first remission consisting of HDCT and ASCT.

Clinical characteristics were analyzed in 140 AML patients diagnosed at a median age of 54 years, including 79 males and 61 females, indicating a sex ratio (m/f) of 1.3. Baseline clinical characteristics were analyzed for the entire cohort and for the three genetic subgroups, with *LAG3* rs870849 encoding isoleucine or threonine at amino acid position 455 of the LAG3 protein (LAG3 I455hom, I455Thet, T455hom) (Table 1). The median age at diagnosis differed in the genetic subgroups with 53–54 years in I455hom and I455Thet, and 58 years in the T455hom subgroups (*p* = 0.51). Anemia was more prevalent in the I455hom subgroup (Hb > 100g/L, 64% vs. 41%, *p* = 0.031). Leukocyte counts differed significantly according to *LAG3* genotype, with higher counts in the I455hom and I455Thet, and lower counts in the T455hom subgroups (median 13 vs. 9 G/L, *p* = 0.03). While leukopenia was more prevalent in the T455hom subgroup (LC < 4G/L, 31% vs. 15%, *p* = 0.015), hyperleukocytosis was more prevalent in the I455hom subgroup (LC > 100G/L, 21% vs. 4%, *p* = 0.05). Platelet counts differed significantly according to *LAG3* genotype, with higher counts in the T455hom subgroup (median 108 vs. 78 G/L, *p* = 0.026). Thrombocytopenia was more prevalent in the I455hom and I455Thet subgroups (PLT < 50G/L, 36% vs. 16%, *p* = 0.05). High LDH levels were more prevalent in the I455Thom subgroup (LDH > 1000U/L, 43% vs. 26%, *p* = 0.06)). Peripheral blast percentage was higher in the I455hom subgroup (median 71% vs. 39%, *p* = 0.008). Bone marrow blast percentage was higher in the I455hom subgroup (median 80 vs. 70%, *p* = 0.19). The FAB classifications varied in the three genetic subgroups, with a prevalence of myelocytic M1 in the I455hom subgroup (39% vs. 14%, *p* = 0.08). Cytogenetic aberrations and somatic mutations were present at balanced proportions in the three genetic subgroups. ELN risk was classified in 140 patients, with 72 (51%) favorable, 30 (21%) intermediate and 38 (27%) adverse risk. The ELN risk classifications varied in the genetic subgroups with a lower proportion of adverse risk in I455hom, and higher proportions of adverse risk in I455Thet and T455hom (18% vs. 30%, *p* = 0.32). The ELN risk distribution in the local AML cohort was significantly different (*p* = 0.008) to the ELN distribution in an unbiased AML cohort with 575 (37%) favorable, 410 (26%) intermediate, and 585 (37%) adverse risk cases [20] (Figure 3).

### 3.5. Clinical Outcomes After HDCT/ASCT According to LAG4 rs870849

**Figure 4:**
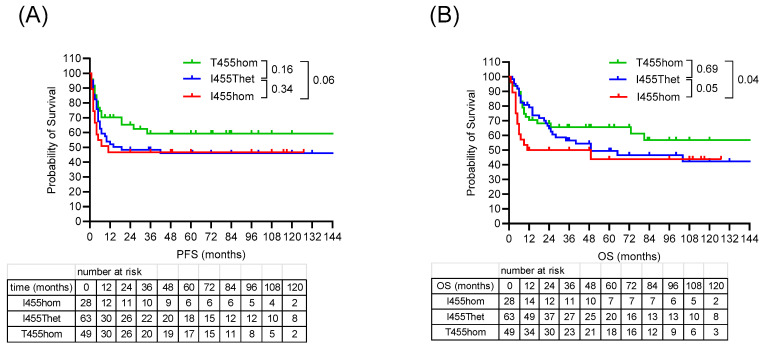
Survival outcomes after HDCT/ASCT according to *LAG3* rs870849 genotypes. Progression-free survival (**A**) and overall survival (**B**) according to *LAG3* genotypes T455hom, I455Thet and I455hom.

The AML patients were treated with standard induction therapy consisting of cytarabine and anthracyline, and consolidation therapy in first remission consisting of high-dose chemotherapy and autologous stem cell transplantation (HDCT/ASCT). Clinical outcomes to HDCT/ASCT differed according to *LAG3* genotype, with one-year PFS rates of 46, 51 and 70% (*p* = 0.06), and one-year OS rates of 50, 78 and 75% (*p* = 0.05) in the I455hom, I455Thet and T455hom subgroups, respectively (Figure 4, Table 2). Clinical outcomes in the I455hom subgroup were characterized by early disease progression and short overall survival, in the I455thet subgroup by early disease progression and prolonged overall survival, and in the T455hom subgroup by prolonged progression-free and overall survival. Complete response (CR) rates differed with 75% CR in the I455hom subgroup and 95% CR in the T455hom and I455Thet subgroups (Table 2). In the multivariate analysis, the *LAG3* variant was not significantly associated with survival outcome, with genetic risk classification, peripheral blast percentage and age as confounding predictors (Table 3).

## 4. Discussion

In this retrospective single-center study, we analyzed the prevalence of the common gene variants *CTLA4* rs231775 and *LAG3* rs870849 in the genome of AML patients, the associated gene risk in the emergence of AML, the associated clinical characteristics, and the associated clinical outcomes. *CTLA4* rs231775 was prevalent at reduced allele frequencies in AML patients, with MAF 0.33 compared to MAF 0.37 in the European population. In contrast, *LAG3* rs870849 was prevalent at elevated allele frequencies in AML patients, with MAF 0.58 compared to MAF 0.39 in the general European population. In all genetic models, *LAG3* rs870849 was a significant AML risk gene, with five times higher risk in T455 homozygote versus I455 homozygote carriers. *LAG3* rs870849 associated with clinical characteristics pertaining to blood count profiles. While leukopenia, moderate anemia and thrombocytopenia were prevalent characteristics in the T455hom subgroup, hyper-leukocytosis, severe anemia and thrombocytopenia prevailed in the I455hom subgroup. LAG3 rs870849 may be a significant common risk allele for AML associated with specific clinical characteristics associated with erythrocyte, leukocyte and thrombocyte imbalances.

Other common risk alleles for AML have been identified in genome-wide association studies (GWAS) including interleukin gene variants IL13 rs1295686 and IL8 rs2227307, growth factor gene variant VEGFA rs25648, histone-methyl-transferase gene variant KMT5B rs4930561 and human leukocyte antigen variant HLA rs3916765 [24,25]. Other AML risk alleles include genes related to autosomal dominant cancer predisposition syndromes (ATM, DDX41, and CHEK2) and to autosomal recessive bone marrow failure syndromes (FANCA, FANCM, SBDS, DNAJC21, and CSF3R) [26,27,28,29]. It was estimated that up to 15% of adult MDS/AML cases are caused by germline predisposition [30].

The effects of *LAG3* rs870849 were dose-dependent in both functions of promoting AML disease development and modulating clinical characteristics, indicating similar dynamics in the molecular mechanisms involved in both processes. The underlying molecular mechanisms may be related to the appropriate balance of T-cell activity and T-cell exhaustion [31,32,33]. The following statements are hypothesis-driven. In AML pathogenesis, *LAG3* rs870849 may increase the number of exhausted T-cells with concomitant loss of immune surveillance. In AML disease, *LAG3* rs870849 may promote T-cell activity and repress leukocyte and leukemic blast cell count. The commonly applied induction therapy may impair the stem cells in AML patients, leading to a reduced reconstitution potential after ASCT, similar to the impaired stem cells in myeloma patients treated with ASCT [34]. *LAG3* rs870849 may promote stem cell function and reconstitution potential after ASCT. The entire complexity of the molecular mechanisms involving different *LAG3* variants remains to be established in future studies.

We previously studied the prevalence and clinical impact of *LAG3* rs870849 in multiple myeloma patients treated with induction therapy and HDCT/ASCT and reported elevated allele frequency and male predominance, higher disease risk and favorable clinical outcomes associated with the germline *LAG3* variant T455hom [10]. Now in this study on *LAG3* rs870849 in AML patients treated with induction therapy and HDCT/ASCT we report similar results, including elevated allele frequency, associated disease risk and favorable clinical outcomes associated with the germline *LAG3* variant T455hom; however, there was no male predominance. The germline variant *LAG3* rs870849 may generally increase the risk of hematological disease, with sex-specific inclination towards multiple myeloma in males, and towards AML in females.

The study was limited by sample size, retrospective single-center design and selection bias, with a majority of patients with favorable risk disease. Due to the small sample size the investigation was explorative, including the risk of false positive and false negative results. The selection bias with a majority of patients with favorable risk disease may have influenced allele frequency and gene risk estimates. The comparison of allele frequencies was made against a large European database rather than a locally matched control cohort. Due to the lack of a control cohort, it was not possible to differentiate between prognostic and predictive value. The presented results may reflect genotype-specific characteristics but should be interpreted cautiously and as hypothesis-generating rather than definitive. Adequately powered case–control studies will be required to formally evaluate the role of *LAG3* rs870849 in AML susceptibility, association with clinical characteristics and clinical impact on treatment outcomes.

## 5. Conclusions

This was a retrospective single-center study on the prevalence and impact of two immune checkpoint germline variants, *LAG3* rs870849 and *CTLA4* rs231775, in AML patients eligible for ASCT. While *LAG3* rs870849 had a higher minor allele frequency in the local AML cohort compared to the European reference population and a dose-dependent increase in AML risk, *CTLA4* rs231775 had a lower minor allele frequency and no associated risk. Baseline blood count profiles differed across *LAG3* genotypes, suggesting a link between *LAG3* rs870849 and levels of erythrocyte/leukocyte/platelet imbalance in AML disease.

## Figures and Tables

**Table 1:** Baseline clinical characteristics according to *LAG3* genotype.

Parameter	I455hom (*n* = 28)	I455het (*n* = 63)	T455hom (*n* = 49)	All Patients (*n* = 140)	*p* *
Male, *n* (%)	16 (57)	36 (57)	27 (55)	79 (56)	0.41
Age, median (range)	54 (24–74)	53 (17–72)	58 (19–70)	54 (17–74)	0.51
Hemoglobin g/L, median (range)	83 (55–135)	88 (38–134)	97 (7–137)	88 (7–137)	0.35
Anemia (Hb < 100g/L), *n* (%)	18 (64)	22 (35)	20 (41)	60 (43)	0.031
Leukocytes G/L, median (range)	13 (1–267)	12 (1–166)	9 (1–272)	11 (1–272)	0.029
Leukopenia (LC < 4 G/L), *n* (%)	4 (15)	11 (16)	18 (31)	30 (19)	0.015
Leukocytosis (LC > 10 G/L), *n* (%)	13 (46)	28 (44)	21 (43)	62 (44)	0.97
Hyperleukocytosis (LC > 100 G/L), *n* (%)	6 (21)	5 (8)	2 (4)	13 (9)	0.05
Platelets G/L, median (range)	78 (8–405)	74 (4–714)	108 (6–268)	85 (4–714)	0.026
Thrombocytopenia (PLT < 50 G/L), *n* (%)	10 (36)	23 (37)	8 (16)	40 (29)	0.05
LDH U/L, median (range)	939 (198–2538)	733 (116–3074)	660 (412–7108)	762 (116–7108)	0.12
LDH >1000 U/L, *n* (%)	12 (43)	15 (24)	13 (26)	40 (29)	0.06
Blast cells PB %, median (range)	71 (7–99)	56 (2–99)	39 (0–94)	50 (0–99)	0.008
Blast cells BM %, median (range)	80 (10–95)	78 (0–100)	70 (25–95)	80 (0–100)	0.19
FAB classification, *n* (%)					0.41
M0	3 (11)	3 (5)	6 (10)	11 (7)	
M1 ^1^	11 (39)	18 (26)	8 (14)	35 (23)	0.08
M2	7 (25)	20 (29)	16 (28)	43 (28)	0.82
M3	1 (3)	0	0	1 (<1)	
M4	6 (22)	17 (24)	13 (22)	36 (23)	0.80
M5	1 (3)	6 (9)	6 (12)	13 (8)	0.49
M6	1 (3)	0	0	1 (<1)	
Pathogenesis					0.82
de novo AML, *n* (%)	25 (89)	58 (92)	43 (92)	126 (90)	
sAML (MDS/MPN-related)*, n* (%)	1 (4)	3 (5)	4 (8)	8 (6)	
tAML (therapy-related), *n* (%)	2 (8)	2 (5)	2 (4)	6 (4)	
Cytogenetic aberrations, *n* (%)					0.97
Normal karyotype	17 (61)	38 (60)	30 (61)	85 (61)	
Abnormal karyotype ^2^	10 (36)	24 (38)	18 (37)	52 (37)	
Complex karyotype	1 (3)	1 (2)	1 (2)	3 (2)	
Somatic mutations, *n* (%)					0.91
normal	7 (25)	12 (17)	14 (24)	33 (21)	
NPM1_mut_ FLT3_wt_ (favorable)	6 (21)	15 (24)	11 (22)	32 (23)	
FLT3-ITD NPM1_wt_ (intermediate)	3 (11)	2 (3)	3 (5)	8 (5)	
FLT3-ITD NPM1_mut_ (intermediate)	4 (14)	10 (14)	6 (10)	20 (13)	
Adverse risk genes ^3^	5 (18)	17 (24)	12 (21)	34 (22)	
Intermediate risk genes ^4^	8 (29)	12 (17)	9 (16)	29 (19)	
ELN-risk classification (2022), *n* (%)					0.32
Favorable (ELN1)	13 (46)	34 (54)	25 (51)	72 (51)	
Intermediate (ELN2)	10 (36)	10 (16)	10 (20)	30 (21)	
Adverse (ELN3)	5 (18)	19 (30)	14 (29)	38 (27)	

PB: peripheral blood, BM: bone marrow; ELN: European LeukemiaNet; FAB: French–American–British; LDH: lactate dehydrogenase; MDS: myelodysplastic syndrome; MPN: myeloproliferative neoplasm; mut: mutated; wt: wildtype; ^1^ includes: AML-M1, AML not further classifiable, AML from MDS (WHO 2008) and AML from MPN (WHO 2008). ^2^ Abnormal karyotypes including t(8;21), INV16, t(9;22)(q34.1;q11.2), t(6;9)(p23.3;q34.1), t(v;11q23.3), t(8;16)(p11.2;p13.3), inv(3)(q21.3q26.2) or t(3;3)(q21.3;q26.2), t(3q26.2;v), −5 or del(5q); −7; −17/abn(17p). ^3^ Adverse-risk genes include ASXL1, BCOR, EZH2, RUNX1, SF3B1, SRFS2, STAG2, U2AF1, TP53, and ZRSR2; ^4^ intermediate-risk genes include DNMT3A, TET2, IDH1, IDH2, KRAS, NRAS, and KIT. * *p* values without Bonferroni correction for multiple testing. Associations are exploratory.

**Table 2:** Clinical outcomes and survival according to *LAG3* genotype, univariate analysis.

Outcomes and Survival	I455hom (*n* = 28)	I455Thet (*n* = 63)	T455hom (*n* = 49)	All patients (*n* = 140)	*p* *
CR, *n* (%)	21 (75)	60 (95)	48 (98)	129 (92)	0.002
Relapse, *n* (%)	11 (39)	32 (51)	16 (33)	59 (42)	0.15
Early relapse, *n* (%)	11 (39)	29 (46)	11 (22)	49 (35)	0.03
One-year PFS, *n* (%)	13 (46)	32 (51)	34 (70)	89 (64)	0.06
Five-year PFS, *n* (%)	12 (43)	30 (48)	30 (61)	72 (52)	0.23
Death, *n* (%)	12 (43)	35 (56)	21 (43)	68 (49)	0.35
Early death, *n* (%)	10 (36)	9 (14)	12 (24)	31 (22)	0.07
One-year OS, *n* (%)	14 (50)	49 (78)	38 (78)	101 (72)	0.05
Five-year OS, *n* (%)	12 (43)	32 (51)	32 (65)	47 (34)	0.12

CR: complete remission; OS: overall survival; PFS: progression-free survival. * Chi-square test.

**Table 3:** Clinical outcomes, multivariate analysis.

Predictors	PFS		OS	
	HR (CI)	*p*	HR (CI)	*p*
rs870849, I455hom vs. T455hom	1.3 (0.6–2.7)	0.48	1.3 (0.6–2.9)	0.59
rs870849, I455hom vs. I455Thet	1.1 (0.5–2.4)	0.81	1.1 (0.5–2.7)	0.81
ELN risk, adverse vs. favorable	1.9 (1.0–3.4)	0.05	2.2 (1.1–4.3)	0.02
ELN risk, intermediate vs. favorable	2.1 (1.1–3.8)	0.02	2.1 (1.1–4.3)	0.03
Age at diagnosis, > 65 vs. <65	1.5 (0.8–3.0)	0.25	2.7 (1.3–5.6)	0.01
Blast cells PB, >50% vs. <50%	1.7 (0.6–5.0)	0.32	1.2 (0.6–2.1)	0.61

ELN: European LeukemiaNet; PB: peripheral blast cells (PB).

## Data Availability

Data available on request due to restrictions, privacy and ethics.

## Abbreviations

The following abbreviations are used in this manuscript: Acute myeloid leukemia (AML), Lymphocyte-activation gene 3 (LAG3), cytotoxic T-lymphocyte associated protein 4 (CTLA4), high-dose chemotherapy (HDCT), autologous stem cell transplant (ASCT), minor allele frequency (MAF).
